# 4,4′-Di-*tert*-butyl-2,2′-dipyridinium dichloride

**DOI:** 10.1107/S1600536811025529

**Published:** 2011-07-06

**Authors:** Tatiana R. Amarante, Isabel S. Gonçalves, Filipe A. Almeida Paz

**Affiliations:** a Department of Chemistry, University of Aveiro, CICECO, 3810-193 Aveiro, Portugal

## Abstract

In the title compound, C_18_H_26_N_2_^2+^·2Cl^−^, the complete dication is generated by crystallographic inversion symmetry; both N atoms are protonated and engaged in strong and highly directional N—H⋯Cl hydrogen bonds. Additional weak C—H⋯Cl contacts promote the formation of a tape along *ca.* [110]. The crystal structure can be described by the parallel packing of these tapes. The crystal studied was a non-merohedral twin with twin law [−1 0 0, 0 −1 0, −0.887 0.179 1] and the final BASF parameter refining to 0.026 (2) .

## Related literature

For metallic complexes of 4,4′-di-*tert*-butyl-2,2′-dipyridyl, see: Momeni *et al.* (2010 ▶); Li *et al.* (2005 ▶). For related organic crystals from our research groups, see: Amarante, Figueiredo *et al.* (2009 ▶); Amarante, Gonçalves & Almeida Paz (2009 ▶); Amarante, Paz *et al.* (2009 ▶); Batsanov *et al.* (2007 ▶); Coelho *et al.* (2007 ▶); Herrmann *et al.* (1990 ▶); Paz & Klinowski (2003 ▶); Paz *et al.* (2002 ▶). For graph-set notation, see: Grell *et al.* (1999 ▶). For a description of the Cambridge Structural Database, see: Allen (2002 ▶). For the refinement, see: Cooper *et al.* (2002 ▶).
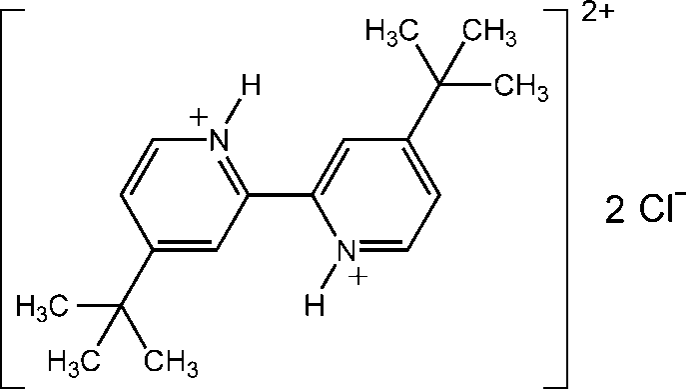


## Experimental

### 

#### Crystal data

C_18_H_26_N_2_^2+^·2Cl^−^
*M* *_r_* = 341.31
Triclinic, 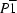

*a* = 5.9017 (8) Å
*b* = 6.1949 (8) Å
*c* = 13.0758 (17) Å
α = 89.633 (8)°
β = 79.049 (7)°
γ = 75.915 (7)°
*V* = 454.84 (10) Å^3^
*Z* = 1
Mo *K*α radiation
μ = 0.36 mm^−1^
*T* = 150 K
0.12 × 0.03 × 0.03 mm

#### Data collection

Bruker X8 KappaCCD APEXII diffractometer
Absorption correction: multi-scan (*SADABS*; Sheldrick, 1998 ▶) *T*_min_ = 0.959, *T*_max_ = 0.989
14551 measured reflections
2054 independent reflections
1654 reflections with *I* > 2σ(*I*)
*R*_int_ = 0.074

#### Refinement

*R*[*F* ^2^ > 2σ(*F* ^2^)] = 0.083
*wR*(*F* ^2^) = 0.188
*S* = 1.25
2054 reflections
107 parameters
1 restraint
H atoms treated by a mixture of independent and constrained refinement
Δρ_max_ = 0.72 e Å^−3^
Δρ_min_ = −0.39 e Å^−3^

### 

Data collection: *APEX2* (Bruker, 2006 ▶); cell refinement: *SAINT-Plus* (Bruker, 2005 ▶); data reduction: *SAINT-Plus*; program(s) used to solve structure: *SHELXTL* (Sheldrick, 2008 ▶); program(s) used to refine structure: *SHELXTL*; molecular graphics: *DIAMOND* (Brandenburg, 2009 ▶); software used to prepare material for publication: *SHELXTL*.

## Supplementary Material

Crystal structure: contains datablock(s) global, I. DOI: 10.1107/S1600536811025529/bt5558sup1.cif

Structure factors: contains datablock(s) I. DOI: 10.1107/S1600536811025529/bt5558Isup2.hkl

Additional supplementary materials:  crystallographic information; 3D view; checkCIF report

## Figures and Tables

**Table 1:** Hydrogen-bond geometry (Å, °)

*D*—H⋯*A*	*D*—H	H⋯*A*	*D*⋯*A*	*D*—H⋯*A*
N1—H1⋯Cl1	0.95 (1)	2.05 (2)	2.967 (4)	162 (5)
C1—H1*A*⋯Cl1^i^	0.95	2.70	3.479 (3)	140
C4—H4*A*⋯Cl1^ii^	0.95	2.61	3.543 (9)	166

Symmetry codes: (i) 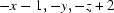; (ii) 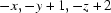.

**Fig. 1.**
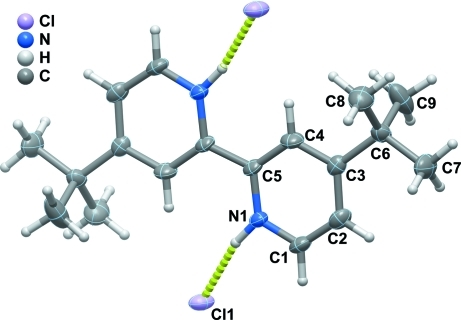
Schematic representation of the molecular units composing the crystal structure of the title compound. Non-hydrogen atoms are represented as displacement ellipsoids drawn at the 70% probability level. Hydrogen atoms are depicted as small spheres with arbitrary radii. The atomic labeling for all non-hydrogen atoms composing the asymmetric unit is provided.

**Fig. 2.**
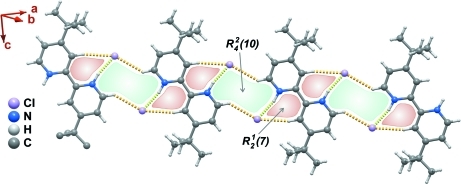
Interconnection of adjacent chloride anions and protonated organic molecules via N—H···Cl and C—H···Cl contacts (green and brown dashed lines, respectively) leading to the formation of a one-dimensional supramolecular tape. For geometrical details on the represented supramolecular contacts see Tables 1 and 2.

**Fig. 3.**
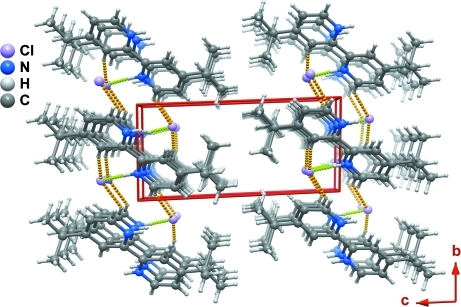
Crystal packing of the title compound viewed in perspective along the [100] direction of the unit cell. N—H···Cl and C—H···Cl intermolecular interactions are represented as green and brown dashed lines, respectively.

## supplementary crystallographic information

### Comment

4,4'-Di-*tert*-butyl-2,2'-dipyridyl is a versatile *N*,*N'*-chelating organic ligand derived from the widely employed 2,2'-bipyridine molecule by the inclusion of two bulky *t*-butyl substituent groups at the 4 and 4' positions. A search in the Cambridge Structural Database (CSD, Version 5.32, November 2010 with three updates) (Allen, 2002) reveals that this molecule forms relatively stable complexes with a large range of metallic cations, including lanthanides, actinides and, mainly, *d*-block cations. Surprisingly, not many crystallographic reports are known in which 4,4'-di-*tert*-butyl-2,2'-dipyridyl is chelated to either *s*- or *p*-block cations: there is a single report in the literature of an organometallic complex with Na^+^ by Li *et al.* (2005), and another very recent with Sn^4+^ by Momeni *et al.* (2010). Concerning organic crystals, besides the crystal structure of 4,4'-di-*tert*-butyl-2,2'-dipyridyl which was recently reported by our group (Amarante & Figueiredo *et al.*, 2009), there is a single crystallographic determination in which this molecule co-crystallizes with hexafluorobenzene (Batsanov *et al.*, 2007). As a continuation of our on-going interest in organic crystals based on pyridine derivatives (Amarante & Gonçalves *et al.*, 2009; Coelho *et al.*, 2007; Paz & Klinowski, 2003; Paz *et al.*, 2002), here we wish to report the crystal structure of the title compound (I) at 150 K, which is an organic salt with chloride anions. Noteworthy, a search in the literature reveals the existence of only one other salt of protonated 4,4'-di-*tert*-butyl-2,2'-dipyridyl moieties, being reported by Herrmann *et al.* (1990) and using perrhenate as the charge-balancing anion.

The asymmetric unit of the title compound is composed of half of a 4,4'-di-*tert*-butyl-2,2'-dipyridinium cation (the molecule has its geometrical centre located over an inversion center) and by a single chloride anion strongly hydrogen bonded to the neighbouring N^+^—H group as depicted in Figure 1. As a consequence, the 4,4'-di-*tert*-butyl-2,2'-dipyridinium cation adopts a typical *trans* conformation around the central C—C bond, very much similar to that observed by us in the crystal structure of the molecule itself (Amarante & Figueiredo *et al.*, 2009) and also by Batsanov *et al.* (2007) in the co-crystal with hexafluorobenzene. This conformation permits a significant reduction of the overall steric repulsion due to the large *tert*-butyl substituent groups.

Each diprotonated organic cation is engaged in a strong and highly directional N^+^—H···Cl^-^ hydrogen bonding interaction with the charge-balancing anions (Table 1 and Figures 1 and 2). These intermolecular connections are further strengthened by the presence of a number of weak C—H···Cl contacts as depicted in Figure 2 (see geometrical details in Table 2), leading to the formation of a supramolecular hydrogen-bonded tape composed of alternating *R^1^_2_(7)* and *R^2^_4_(10)* graph set motifs (Grell *et al.*, 1999). The crystal structure of the title compound is obtained by the close packing of these supramolecular tapes as shown in Figure 3.

### Experimental

Irregular, poorly-formed crystals of the title compound were isolated as a minor secondary product during the preparation of the oxodiperoxo complex MoO(O_2_)_2_(tbbpy) (where tbbpy stands for 4,4'-di-*tert*-butyl-2,2'-dipyridyl) previously reported by our group (Amarante & Paz *et al.*, 2009).

### Refinement

Hydrogen atoms bound to carbon have been placed at their idealized positions and were included in the final structural model in riding-motion approximation with C—H distances of 0.95 Å (aromatic C—H) and 0.98 Å (terminal —CH_3_ groups). The hydrogen atom bound to the nitrogen atom was directly located from difference Fourier maps and was included in the final structural model with the N—H distance restrained to 0.95 Å. The isotropic displacement parameters for these hydrogen atoms were fixed at 1.2 (for the former family of hydrogen atoms) or 1.5×*U*_eq_ (for the two latter families) of the respective parent atoms.

The final structural refinement was performed by using the twin law [-1 0 0, 0 - 1 0, -0.887 0.179 1] (Cooper *et al.*, 2002) with the final BASF parameter refining to 0.026 (2).

### Crystal data

**Table:**

C_18_H_26_N_2_^2+^·2Cl^−^	*Z* = 1
*M**_r_* = 341.31	*F*(000) = 182
Triclinic, *P*1	*D*_x_ = 1.246 Mg m^−^^3^
Hall symbol: -P 1	Mo *K*α radiation, λ = 0.71073 Å
*a* = 5.9017 (8) Å	Cell parameters from 3784 reflections
*b* = 6.1949 (8) Å	θ = 3.2–28.8°
*c* = 13.0758 (17) Å	µ = 0.36 mm^−^^1^
α = 89.633 (8)°	*T* = 150 K
β = 79.049 (7)°	Block, colourless
γ = 75.915 (7)°	0.12 × 0.03 × 0.03 mm
*V* = 454.84 (10) Å^3^	

### Data collection

**Table:**

Bruker X8 KappaCCD APEXII diffractometer	2054 independent reflections
Radiation source: fine-focus sealed tube	1654 reflections with *I* > 2σ(*I*)
graphite	*R*_int_ = 0.074
ω and φ scans	θ_max_ = 27.5°, θ_min_ = 3.6°
Absorption correction: multi-scan (*SADABS*; Sheldrick, 1998)	*h* = −7→7
*T*_min_ = 0.959, *T*_max_ = 0.989	*k* = −8→8
14551 measured reflections	*l* = −16→16

### Refinement

**Table:**

Refinement on *F*^2^	Primary atom site location: structure-invariant direct methods
Least-squares matrix: full	Secondary atom site location: difference Fourier map
*R*[*F*^2^ > 2σ(*F*^2^)] = 0.083	Hydrogen site location: inferred from neighbouring sites
*wR*(*F*^2^) = 0.188	H atoms treated by a mixture of independent and constrained refinement
*S* = 1.25	*w* = 1/[σ^2^(*F*_o_^2^) + (0.*P*)^2^ + 2.3813*P*] where *P* = (*F*_o_^2^ + 2*F*_c_^2^)/3
2054 reflections	(Δ/σ)_max_ < 0.001
107 parameters	Δρ_max_ = 0.72 e Å^−^^3^
1 restraint	Δρ_min_ = −0.39 e Å^−^^3^

### Special details

**Table:**

Geometry. All e.s.d.'s (except the e.s.d. in the dihedral angle between two l.s. planes) are estimated using the full covariance matrix. The cell e.s.d.'s are taken into account individually in the estimation of e.s.d.'s in distances, angles and torsion angles; correlations between e.s.d.'s in cell parameters are only used when they are defined by crystal symmetry. An approximate (isotropic) treatment of cell e.s.d.'s is used for estimating e.s.d.'s involving l.s. planes.
Refinement. Refinement of *F*^2^ against ALL reflections. The weighted *R*-factor *wR* and goodness of fit *S* are based on *F*^2^, conventional *R*-factors *R* are based on *F*, with *F* set to zero for negative *F*^2^. The threshold expression of *F*^2^ > σ(*F*^2^) is used only for calculating *R*-factors(gt) *etc*. and is not relevant to the choice of reflections for refinement. *R*-factors based on *F*^2^ are statistically about twice as large as those based on *F*, and *R*- factors based on ALL data will be even larger.

### Fractional atomic coordinates and isotropic or equivalent isotropic displacement parameters (Å^2^)

**Table:**

	*x*	*y*	*z*	*U*_iso_*/*U*_eq_	
Cl1	−0.5222 (2)	0.2342 (2)	1.16162 (10)	0.0234 (3)	
N1	−0.1611 (7)	0.2931 (6)	0.9791 (3)	0.0167 (8)	
H1	−0.253 (8)	0.281 (9)	1.046 (2)	0.025*	
C1	−0.1874 (8)	0.1531 (8)	0.9071 (4)	0.0189 (10)	
H1A	−0.2924	0.0589	0.9262	0.023*	
C2	−0.0648 (8)	0.1433 (8)	0.8057 (4)	0.0195 (10)	
H2A	−0.0832	0.0419	0.7555	0.023*	
C3	0.0868 (8)	0.2843 (7)	0.7777 (4)	0.0164 (9)	
C4	0.1157 (8)	0.4233 (8)	0.8556 (4)	0.0196 (10)	
H4A	0.2225	0.5166	0.8387	0.024*	
C5	−0.0083 (8)	0.4275 (7)	0.9569 (3)	0.0150 (9)	
C6	0.2115 (9)	0.3004 (8)	0.6655 (4)	0.0186 (10)	
C7	0.1739 (10)	0.1275 (9)	0.5916 (4)	0.0295 (12)	
H7A	0.2402	−0.0225	0.6134	0.044*	
H7B	0.2543	0.1459	0.5204	0.044*	
H7C	0.0031	0.1484	0.5935	0.044*	
C8	0.4802 (10)	0.2668 (10)	0.6597 (4)	0.0303 (12)	
H8A	0.5076	0.3804	0.7045	0.045*	
H8B	0.5581	0.2801	0.5876	0.045*	
H8C	0.5466	0.1185	0.6834	0.045*	
C9	0.1040 (12)	0.5355 (9)	0.6314 (4)	0.0358 (14)	
H9A	−0.0690	0.5593	0.6396	0.054*	
H9B	0.1730	0.5500	0.5581	0.054*	
H9C	0.1396	0.6468	0.6746	0.054*	

### Atomic displacement parameters (Å^2^)

**Table:**

	*U*^11^	*U*^22^	*U*^33^	*U*^12^	*U*^13^	*U*^23^
Cl1	0.0211 (6)	0.0222 (6)	0.0284 (6)	−0.0127 (4)	0.0011 (5)	−0.0006 (4)
N1	0.0168 (19)	0.0172 (18)	0.020 (2)	−0.0089 (15)	−0.0062 (15)	0.0031 (15)
C1	0.018 (2)	0.016 (2)	0.027 (2)	−0.0082 (18)	−0.0080 (19)	0.0013 (18)
C2	0.019 (2)	0.014 (2)	0.026 (3)	−0.0034 (18)	−0.0075 (19)	−0.0018 (18)
C3	0.014 (2)	0.014 (2)	0.020 (2)	−0.0001 (17)	−0.0051 (18)	−0.0012 (17)
C4	0.016 (2)	0.022 (2)	0.024 (2)	−0.0095 (19)	−0.0028 (19)	0.0025 (19)
C5	0.012 (2)	0.015 (2)	0.021 (2)	−0.0048 (17)	−0.0073 (17)	0.0014 (18)
C6	0.023 (2)	0.017 (2)	0.017 (2)	−0.0079 (19)	−0.0023 (19)	0.0010 (17)
C7	0.034 (3)	0.032 (3)	0.021 (3)	−0.012 (2)	0.002 (2)	−0.009 (2)
C8	0.023 (3)	0.040 (3)	0.028 (3)	−0.013 (2)	0.000 (2)	−0.002 (2)
C9	0.052 (4)	0.025 (3)	0.022 (3)	0.001 (3)	−0.001 (3)	0.005 (2)

### Geometric parameters (Å, °)

**Table:**

N1—C1	1.340 (6)	C6—C7	1.530 (7)
N1—C5	1.361 (5)	C6—C8	1.536 (7)
N1—H1	0.952 (10)	C6—C9	1.541 (7)
C1—C2	1.378 (7)	C7—H7A	0.9800
C1—H1A	0.9500	C7—H7B	0.9800
C2—C3	1.396 (6)	C7—H7C	0.9800
C2—H2A	0.9500	C8—H8A	0.9800
C3—C4	1.399 (6)	C8—H8B	0.9800
C3—C6	1.526 (6)	C8—H8C	0.9800
C4—C5	1.383 (6)	C9—H9A	0.9800
C4—H4A	0.9500	C9—H9B	0.9800
C5—C5^i^	1.478 (9)	C9—H9C	0.9800
			
C1—N1—C5	122.0 (4)	C3—C6—C9	106.8 (4)
C1—N1—H1	113 (3)	C7—C6—C9	109.1 (4)
C5—N1—H1	125 (3)	C8—C6—C9	109.9 (4)
N1—C1—C2	121.2 (4)	C6—C7—H7A	109.5
N1—C1—H1A	119.4	C6—C7—H7B	109.5
C2—C1—H1A	119.4	H7A—C7—H7B	109.5
C1—C2—C3	119.1 (4)	C6—C7—H7C	109.5
C1—C2—H2A	120.4	H7A—C7—H7C	109.5
C3—C2—H2A	120.4	H7B—C7—H7C	109.5
C2—C3—C4	118.1 (4)	C6—C8—H8A	109.5
C2—C3—C6	122.7 (4)	C6—C8—H8B	109.5
C4—C3—C6	119.1 (4)	H8A—C8—H8B	109.5
C5—C4—C3	121.2 (4)	C6—C8—H8C	109.5
C5—C4—H4A	119.4	H8A—C8—H8C	109.5
C3—C4—H4A	119.4	H8B—C8—H8C	109.5
N1—C5—C4	118.3 (4)	C6—C9—H9A	109.5
N1—C5—C5^i^	117.1 (5)	C6—C9—H9B	109.5
C4—C5—C5^i^	124.6 (5)	H9A—C9—H9B	109.5
C3—C6—C7	112.4 (4)	C6—C9—H9C	109.5
C3—C6—C8	110.2 (4)	H9A—C9—H9C	109.5
C7—C6—C8	108.5 (4)	H9B—C9—H9C	109.5
			
C5—N1—C1—C2	−1.8 (7)	C3—C4—C5—N1	−0.3 (7)
N1—C1—C2—C3	−0.9 (7)	C3—C4—C5—C5^i^	−178.7 (5)
C1—C2—C3—C4	2.8 (7)	C2—C3—C6—C7	−6.6 (6)
C1—C2—C3—C6	−174.2 (4)	C4—C3—C6—C7	176.5 (4)
C2—C3—C4—C5	−2.2 (7)	C2—C3—C6—C8	−127.7 (5)
C6—C3—C4—C5	174.9 (4)	C4—C3—C6—C8	55.4 (6)
C1—N1—C5—C4	2.4 (6)	C2—C3—C6—C9	113.0 (5)
C1—N1—C5—C5^i^	−179.1 (5)	C4—C3—C6—C9	−64.0 (6)

Symmetry codes: (i) −*x*, −*y*+1, −*z*+2.

### Hydrogen-bond geometry (Å, °)

**Table:**

*D*—H···*A*	*D*—H	H···*A*	*D*···*A*	*D*—H···*A*
N1—H1···Cl1	0.95 (1)	2.05 (2)	2.967 (4)	162 (5)
C1—H1A···Cl1^ii^	0.95	2.70	3.479 (3)	140
C4—H4A···Cl1^i^	0.95	2.61	3.543 (9)	166

Symmetry codes: (ii) −*x*−1, −*y*, −*z*+2; (i) −*x*, −*y*+1, −*z*+2.
